# ICTV Virus Taxonomy Profile: *Quadriviridae*

**DOI:** 10.1099/jgv.0.001152

**Published:** 2018-09-28

**Authors:** Sotaro Chiba, José R. Castón, Said A. Ghabrial, Nobuhiro Suzuki, ICTV Report Consortium

**Affiliations:** ^1^​ Asian Satellite Campuses Institute, Nagoya University, Nagoya 464-0861, Japan; ^2^​ Department of Structure of Macromolecules, Centro Nacional de Biotecnología (CNB-CSIC), Cantoblanco, Madrid 28049, Spain; ^3^​ Department of Plant Pathology, University of Kentucky, Lexington, KY 40546, USA; ^4^​ Institute of Plant Science and Resources, Okayama University, Chuo 2-20-1, Kurashiki 7100046, Japan

**Keywords:** *Quadriviridae*, ICTV Report, taxonomy

## Abstract

The *Quadriviridae* is a monogeneric family of non-enveloped spherical viruses with quadripartite dsRNA genomes, each segment of 3.5–5.0 kbp, comprising 16.8–17.1 kbp in total. The family includes the single species *Rosellinia necatrix quadrivirus 1*. All quadriviruses infect filamentous fungi, and have unique virion structures compared with other known dsRNA viruses. Pathogenicity has not been reported for these viruses. This is a summary of the ICTV Report on the taxonomy of family *Quadriviridae*, which is available at http://www.ictv.global/report/quadriviridae.

## Abbreviations

CP, Capsid protein; RdRP, RNA-dependent RNA polymerase.

## Virion

**Fig. 1.**
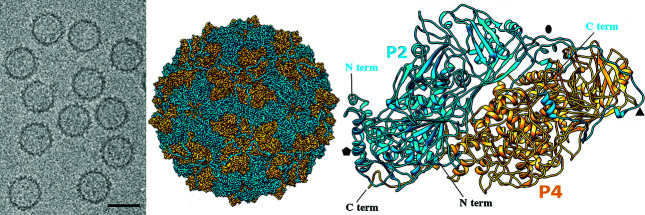
Quadrivirus particle structure. (Left) Cryo-EM image of Rosellinia necatrix quadrivirus 1-W1118 (scale bar, 50 nm). (Middle) Cryo-EM map of the Rosellinia necatrix quadrivirus 1 capsid viewed along a twofold axis, showing P2 (blue) and P4 (yellow) proteins. (Right) Atomic model of P2 (blue) and P4 (yellow) (top view) at a resolution of 3.7 Å. N and C termini are indicated. Symbols indicate icosahedral symmetry axes (from [2]).

Quadriviruses form rigid spherical particles of 45 nm in diameter (Table 1, Fig. 1). Each capsid consists of 60 copies of heterodimers of two major structural proteins, P2 and P4 (Fig. 1). The P2 and P4 proteins form an asymmetric hetero-dimer subunit, and 60 of those build the *T=1* capsid [1]. The P2 and P4 proteins of the Rosellinia necatrix quadrivirus 1 strains W1075 and W1118 are more than 80 % identical. While P2–P4 heterodimers are organized similarly to homodimers of other spherical dsRNA viruses, P2 and P4 have acquired domains situated on the outer surface that are hypothesized to possess actin regulatory (P2-domain) and protease (P4-domain) enzyme activities [2]. The transfection competency of purified virions has not been demonstrated.

**Table 1.** Characteristics of the family *Quadriviridae*

Typical member:	Rosellinia necatrix quadrivirus 1-W1075, (RNA1: AB620061; RNA2: AB620062; RNA3: AB620063; RNA4: AB620064), species *Rosellinia necatrix quadrivirus 1*, genus *Quadrivirus*
Genome	Four, linear dsRNAs of 3.5–5.0 kbp, 16.8–17.1 kbp in total
Virion	Isometric, non-enveloped, 45 nm in diameter; dsRNA segments may be separately encapsidated
Replication	No information available
Translation	Presumed from monocistronic positive-sense transcripts of each genomic dsRNA
Host range	Fungi
Taxonomy	One genus including a single species

## Replication

Each dsRNA is monocistronic. The RNA-dependent RNA polymerase is believed to function as both a transcriptase and a replicase, with end-to-end transcription from dsRNA segments expected. Neither in-particle replication nor transcription has been reported, but both are highly likely.

## Genome

**Fig. 2.**
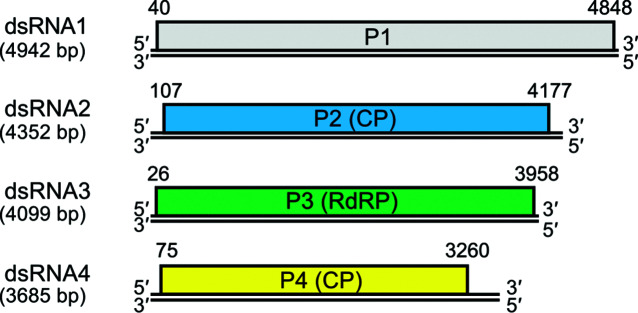
Genome organization of Rosellinia necatrix quadrivirus 1-W1075. dsRNA2 and dsRNA4 encode structural proteins (capsid protein, CP) while dsRNA3 encodes the replicase (RNA-dependent RNA polymerase, RdRP). dsRNA1 encodes a non-structural protein of unknown function. Double lines indicate genomic dsRNAs; boxes indicate open reading frames on the positive-sense strands which begin and end at the nucleotide positions indicated.

The four genome segments, termed dsRNA1–4, are approximately 3.5–5.0 kbp. Whether the positive-sense transcripts of the genomic dsRNAs are capped at their 5′-terminal ends, and whether they are polyadenylated at the 3′-terminal ends, remain unknown (Fig. 2). The dsRNA3 encodes an RNA-dependent RNA polymerase (P3), while dsRNA2 and dsRNA4 encode major structural proteins, the capsid proteins (CP) P2 and P4, respectively [3, 4]. The longest genome segment, dsRNA1, encodes a polypeptide (P1) of unknown function. ‘CAA’ tri-nucleotide repeats are found at the 5′-untranslated region and adjacent coding regions, as in the case of chrysoviruses [5] and certain partitiviruses [6]. The ‘CAA’ repeat is considered to be a translational enhancer, as in the case of fungal chrysoviruses. Internal ribosomal entry sites reside in the 5′-untranslated region of chrysoviruses, but not in quadriviruses [7]. The replication, transcription and translation strategies of viral dsRNAs have not been explored. The extreme terminal nucleotides of dsRNA segments are heterogeneous; the 5′- and 3′-ends of coding strands are ‘C or U’ and ‘G or A’, respectively [3, 4].

## Taxonomy

The family *Quadriviridae*, which was established in 2013, includes the single genus *Quadrivirus* with a single species *Rosellinia necatrix quadrivirus 1*. This species includes two fully sequenced viruses, the exemplar isolate Rosellinia necatrix quadrivirus 1-W1075, and Rosellinia necatrix quadrivirus 1-W1118, as well as the partially characterized strain Rosellinia necatrix quadrivirus 1-W726, all isolated from the filamentous ascomycetous fungus *Rosellinia necatrix* that is infectious to over 400 plants [8]. Members of the family are phylogenetically more closely related to members of the family *Totiviridae* (genus *Totivirus*; monopartite dsRNA genome) than known quadripartite viruses such as members of the family *Chrysoviridae* and other unclassified quadripartite viruses. Large dsRNAs have been reported associated with Amasya cherry disease that encode RNA-dependent RNA polymerases related to those of quadriviruses, although little is known about the biology of the virus [9]. While quadriviruses persistently infect their fungal hosts, they are distributed unevenly within a fungal colony [10]. No symptomatic infection has been reported [3, 4].

## Resources

Full ICTV Online (10th) Report: www.ictv.global/report/quadriviridae.
